# TRADITIONAL COMPLEMENTARY AND ALTERNATIVE MEDICINE USE OF KOREAN AMERICAN OLDER ADULTS: CULTURAL AND HEALTH NEEDS

**DOI:** 10.1093/geroni/igae098.3757

**Published:** 2024-12-31

**Authors:** Grace Yi, Yuri Jang, Leah Martinez

**Affiliations:** California State University Fullerton, Fullerton, California, United States; University of Southern California, Los Angeles, California, United States; California State University, Fullerton, California State University, Fullerton, California, United States

## Abstract

The Korean American population, primarily comprised of first-generation immigrants, is rapidly growing and aging. Literature underscores the significant challenges faced by KAs, largely due to limited English proficiency and cultural differences, which impact their accessibility to the healthcare system and, as a result, health disparity. This study examines the use of Traditional Complementary and Alternative Medicine (TCAM), such as acupuncture and traditional herbal medicines, as a culturally appropriate means to address health inequality. Using data from the Study of Older Korean Americans, a multisite survey conducted in 2017-2018 across five states (California, New York, Texas, Hawaii, and Florida), this study analyzed factors influencing TCAM use among Korean American older adults (KAOA) aged 60 and above (N=2,176) through logistic regression analysis using StataBE18. Over one-quarter (27.2%) of participants reported TCAM use. After controlling demographic factors (age, gender, marital status, education, financial status, and spirituality), significant predictors of TCAM use included acculturation factors, such as ethnic culture closeness (Odds Ratio [OR]=1.04, p<.05), the need for a translator in healthcare settings (OR=.69, p<.01), and residing in areas with a high density of Korean Americans (OR=1.40, p<.05). Also, health and mental health-related factors included cancer (OR=.53, p<.05), arthritis (OR=1.69, p<.001), needing help with daily activities (OR=1.10, p<.05), and experiencing a lack of interest in life (OR=1.72, p<.05). These findings highlight the significance of acculturation and health/mental health factors in determining TCAM use among KAOA and call for health policymakers and providers to adopt culturally competent approaches when working with ethnic minority older adults.

